# The Cross-Sectional and Longitudinal Associations Between Adherence to 24-Hour Movement Behavior Guidelines and Bone Health in Young Children

**DOI:** 10.3390/healthcare12212173

**Published:** 2024-10-31

**Authors:** Dan Li, Lifang Zhang, Ting Hu, Jiameng Ma, Xianxiong Li, Xiang Zhang, Hyunshik Kim

**Affiliations:** 1 School of Physical Education, Hunan Normal University, Changsha 410012, China; lidan97@hunnu.edu.cn (D.L.); hutingting728@hunnu.edu.cn (T.H.); lixianxiong@hunnu.edu.cn (X.L.); 2 School of Sport Science, Changsha Normal University, Changsha 410100, China; zhanglifang@csnu.edu.cn; 3 Faculty of Sports Science, Sendai University, Shibata 9891693, Japan; jm-ma@sendai-u.ac.jp; 4 Physical Education & Sports Science, National Institute of Education, Nanyang Technological University, Singapore 637616, Singapore

**Keywords:** 24-hour movement behavior, bone health, bone mineral density, young children

## Abstract

Background: Adherence to the World Health Organization’s 24-hour movement behavior (24-HMB) guidelines is associated with various health outcomes. However, its relationship with bone mineral density (BMD) in young children has not been investigated. This study aimed to examine the cross-sectional and longitudinal associations between adherence to the 24-HMB guidelines and BMD in young children. Methods: A total of 120 children aged 3–5 years were recruited from three kindergartens in Changsha, Hunan, China. Physical activity (PA) was objectively measured using ActiGraph wGT3X-BT accelerometers, and BMD was assessed using the Sunlight Omnisense 7000P ultrasound device. Screen time (ST) and sleep duration (SD) were reported by parents. Logistic regression was used to analyze the associations between guideline adherence and BMD. Results: Only 5.5% of the participants met all three guidelines for PA, ST, and SD, while 16.5% did not meet any. In the cross-sectional analysis, young children who met both ST and SD guidelines (OR = 0.29, 95% CI: 0.09, 0.95) had a lower risk of insufficient BMD compared to those who met none. In the one-year cohort follow-up, young children who met the PA guideline at baseline (OR = 0.22, 95% CI: 0.07, 0.71), as well as those who met both the PA and ST guidelines (OR = 0.18, 95% CI: 0.04, 0.83) or all three (OR = 0.13, 95% CI: 0.03, 0.69), had a lower risk of insufficient BMD at one year. Conclusions: Adherence to the 24-HMB guidelines may promote bone health in young children. Future research should prioritize achievable goals, such as limiting ST and ensuring adequate SD, while gradually increasing MVPA to optimize bone development.

## 1. Introduction

Although osteoporosis predominantly affects individuals in later life, its origins can be traced back to early childhood [1]. Bone development during childhood is critical, as approximately 50% of adult bone mass is accumulated by the end of adolescence [2]. Achieving optimal peak bone mass during childhood is key to reducing the risk of osteoporosis and other bone conditions in adulthood [3]. Research indicates that insufficient bone mineral density (BMD) in early childhood increases the risk of poor bone health outcomes, such as bone fragility and fractures, later in life [4,5].

Young children’s BMD is influenced by multiple factors, such as genetics, nutrition, and diet. A substantial proportion of the variance in BMD is explained by genetics and is therefore not modifiable [6]. Adequate calcium and vitamin D intake is essential for bone mineralization, with higher intakes linked to better BMD outcomes [7]. Diets rich in fruits, vegetables, and whole grains also provide key nutrients like magnesium, phosphorus, and vitamin K, which support bone health [8]. Additionally, engaging in healthy daily movement behaviors, including adequate physical activity (PA), limited screen time (ST), and sufficient sleep duration (SD), is also considered crucial for bone health.

PA plays a well-established role in promoting bone mineral density in young children [9]. Weight-bearing activities, such as running, jumping, and playing ball games, generate mechanical loads that stimulate bone formation and enhance bone strength [10]. Studies have consistently shown that young children who engage in higher levels of PA tend to have better bone health compared to their less active peers [11]. Specifically, moderate-to-vigorous physical activity (MVPA) has been linked to increased bone mass and density in early childhood [12]. Conversely, excessive ST may have a negative impact on bone health [13,14]. Research indicates that increasing durations of ST might be a risk factor for bone stiffness index development [15]. Adequate SD is another important factor influencing bone health in young children [16,17]. Studies have indicated that young children with regular and sufficient SD tend to exhibit better bone health outcomes, while poor sleep quality or insufficient SD has been linked to adverse effects on bone mass accrual [18].

In recent years, there has been a significant shift in research focus, emphasizing the importance of considering PA, ST, and SD together rather than in isolation [19,20]. While each behavior independently influences health outcomes, their combined effects may reveal interactions that are not apparent when studied separately [21,22,23]. Recognizing the interdependence of these behaviors, recent guidelines have adopted a comprehensive 24-hour movement behavior (24-HMB) framework. These guidelines recommend a balanced approach to health, encompassing the entire day’s activity patterns, including (i) engaging in at least 180 min of PA, of which at least 60 min is MVPA, (ii) limiting recreational ST to no more than 60 min, and (iii) ensuring 10–13 h of SD [24]. Numerous studies have demonstrated that adherence to the 24-HMB guidelines is associated with various health outcomes in young children, including body mass index (BMI) [25,26], cognitive function [27,28], emotional behavior [29,30], fundamental motor skills [31,32], and physical fitness [33,34]. However, no studies to date have examined whether adherence to the 24-HMB guidelines is significantly associated with BMD in young children, as it is with other established health indicators. A study from China investigated the interactive effects of PA and SD on BMD in young children [17]; however, it did not include ST, limiting our understanding of the comprehensive relationship between PA, ST, and SD with BMD. Moreover, this study was cross-sectional, precluding any inference of potential causal relationships.

Therefore, this study aims to investigate the cross-sectional and longitudinal associations between adherence to the 24-HMB guidelines and BMD in young children, providing deeper insights into the potential benefits of 24-HMB adherence for early bone health.

## 2. Materials and Methods

### 2.1. Study Design

A cross-sectional and longitudinal design was employed. Cross-sectional data were collected from kindergartens between September and November 2022 to examine whether adherence to the 24-HMB guidelines is associated with BMD at baseline. Longitudinal data were then collected between September and November 2023 in the same kindergartens, to assess whether baseline adherence to the 24-HMB guidelines is associated with BMD one year later.

### 2.2. Participants and Procedures

Between September and November 2022, recruitment notices were posted through posters and class presentations in three kindergartens in Changsha, Hunan, China, to recruit 3- to 5-year-old children without congenital developmental issues for the study. During parent meetings, detailed explanations of the study’s purpose, content, and procedures were provided to parents who expressed interest in their child’s participation. One week after the parent meetings, the parents who agreed to their child’s participation provided written consent. The study procedures were explained to the young children using age-appropriate language, and they were given the opportunity to decline participation. Once both parents and children agreed, the parents provided proxy reports on their child’s ST, SD, and relevant demographic information. PA and BMD were measured using the ActiGraph wGT3X-BT accelerometer and the Israeli Sunlight Omnisense 7000P ultrasound device, respectively. One year later, the BMD of the baseline participants was remeasured using the Israeli Sunlight Omnisense 7000P ultrasound device. The research complied with the ethical principles outlined in the Declaration of Helsinki and was approved by the Ethics Committee of Hunan Normal University (approval number: 2022-334).

The required sample size for this study was estimated using G*Power 3.1. Based on previous research [35], the prevalence of insufficient BMD in young children was estimated at 29.3% [36]. An effect size of 2.0, an alpha level (α) of 0.05, and a statistical power (1 − β) of 0.80 were set for the calculation. The required sample size was determined to be 93 participants. To allow for potential dropout or loss to follow-up, a 20% increase was applied, resulting in a target sample size of 112 participants.

**Figure 1:**
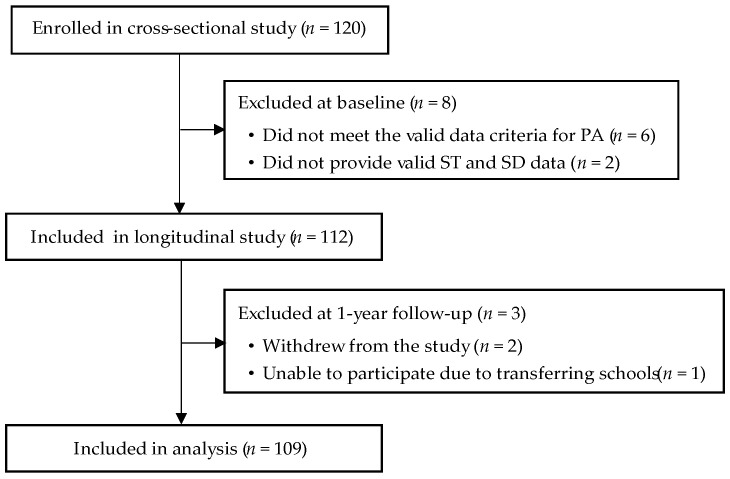
Details about cleaning invalid and missing data in this study. Abbreviations: PA—Physical Activity; ST—Screen Time; SD—Sleep Duration.

A total of 120 children aged 3–5 participated in the study from September to November 2022. Of these, 6 children did not meet the valid data criteria for PA, and 2 parents did not provide ST and SD data, resulting in 112 valid baseline data sets. During the follow-up period, BMD was remeasured in the 112 baseline participants. However, two children declined to continue participation, and one child transferred to another school, making it impossible to collect BMD data. Ultimately, 109 valid data sets were obtained. Details about cleaning invalid and missing data in this study are presented in Figure 1.

### 2.3. Measurement

#### 2.3.1. PA

PA was measured using a triaxial accelerometer, the ActiGraph wGT3X-BT, which has been validated for assessing PA levels in young children with reasonable accuracy [37,38]. Trained research staff placed the ActiGraph wGT3X-BT on the right hip of each preschool child using an elastic waistband. Parents were instructed to ensure their child wore the device for 7 consecutive days, removing it only during water-based activities such as bathing or swimming, and during sleep. The device was set to sample at 60 Hz and recorded data in 15 s epochs [39]. Non-wear time was defined using the Choi algorithm [40]. PA data were considered valid if the participant wore the device for at least 3 days (including 1 weekend day), with a minimum wear time of 10 h per day [41]. PA intensity was classified using the cut-points established by Butte et al. to distinguish between light-intensity physical activity (LPA) and MVPA [42]. Young children who achieved ≥ 60 min/day of MVPA were classified as meeting PA guidelines.

#### 2.3.2. ST

ST was measured by asking parents to report the hours their children spent on various screen-based activities on weekdays and weekends over the past week, including reading e-books, using educational software, watching educational videos, playing mobile games, watching short videos, and using tablets. The average daily ST was calculated using the following formula: [(ST on weekdays × 5) + (ST on weekends × 2)]/7 [29]. Young children with ST ≤ 1 h/day were classified as meeting the ST guideline.

#### 2.3.3. SD

Parents were asked to report their children’s bedtime and wake-up time on weekdays and weekends over the previous week. The SD was calculated using bedtime and wake-up time. The average SD was computed using the following formula: [(SD on weekdays × 5) + (SD on weekends × 2)]/7 [29]. Young children with an SD of 10–13 h/day were classified as meeting the SD guideline.

#### 2.3.4. BMD

At baseline and one year later, young children’s BMD was assessed by measuring the speed of sound (SOS) at the midshaft of the non-dominant tibia using the Israeli Sunlight Omnisense 7000P ultrasound device [43,44]. The validity of this device has been confirmed through clinical studies [45]. The results were expressed as z-scores, calculated as Z-score = (measured BMD value − mean BMD of the same age, sex, and race)/standard deviation of the same age, sex, and race [46]. A z-score > −1 was classified as normal BMD, while a z-score ≤ −1 was considered indicative of insufficient BMD [36].

#### 2.3.5. Covariates

Following the existing literature, several potential control variables were identified, including age, sex assigned at birth, height, weight, BMI, and family socio-economic status (SES). The age and sex of the young children were reported by parents. Height and weight were measured by trained researchers using a stadiometer and a weight scale. BMI was calculated using the formula: weight (kg)/height (m^2^). BMI percentiles were then determined using the World Health Organization growth standards [47], with underweight defined as a BMI < 5th percentile, normal weight as a BMI ≥ 5th but <85th percentile, overweight as a BMI ≥ 85th but <95th percentile, and obesity as a BMI ≥ 95th percentile. Parental education level, occupation, and family income were reported by parents, and family SES was calculated via principal component analysis of parental education, parental occupation, and family income [48,49]. Family SES scores were divided into tertiles, with the lowest scoring 33% of families classified as low SES, the highest 33% as high SES, and the remaining families classified as medium SES.

### 2.4. Statistical Analysis

All statistical analyses were performed using SPSS version 27.0. Descriptive analyses were conducted to describe the characteristics of the sample. Quantitative data were assessed for normality using the Shapiro–Wilk test and presented as mean ± standard deviation if normally distributed. Categorical data were presented as numbers and percentages. Logistic regression models were used to estimate the odds ratios (ORs) for assessing the cross-sectional association between adherence to the 24-HMB guidelines and the risk of insufficient BMD, as well as the longitudinal association one year later. The number of 24-HMB guidelines met and specific combinations of the guidelines were included as independent variables in the models. All regression models were adjusted for age, sex, height, weight, BMI, and family SES. The significance level for all statistical tests was set at α ≤ 0.05.

## 3. Results

### 3.1. Participant Characteristics and Adherence to 24-HMB Recommendations

The sample characteristics are presented in Table 1. Among the 109 participants, the baseline mean age was 4.3 ± 0.6 years, and 44.0% were girls. The average height and weight were 107.4 cm and 18.4 kg, respectively. In terms of BMI, 80.7% were classified as normal. Additionally, 56.0% of the young children came from high-SES families. On average, young children spent 188.0 ± 40.6 min/day in LPA, 46.7 ± 20.6 min/day in MVPA, 100.1 ± 91.7 min/day in ST, and 11.8 ± 1.7 h/day in SD at baseline. There was no significant difference in the proportion of participants with insufficient BMD between baseline and the 1-year follow-up (*X*^2^ = 0.747, *p* = 0.388 > 0.05).

**Figure 2:**
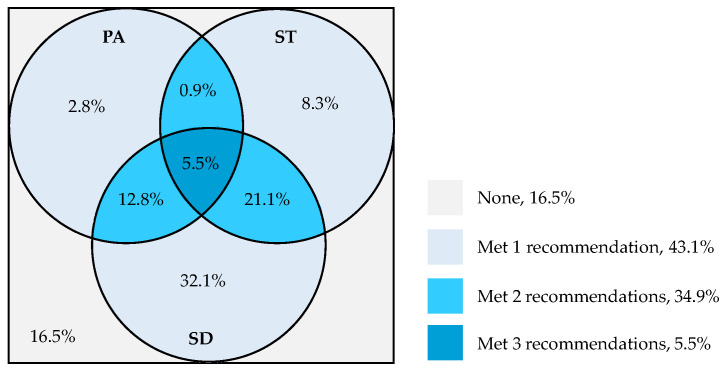
Proportion (%) of young children meeting each of the 24-HMB guidelines at baseline (*n* = 109). Abbreviations: PA—Physical Activity; ST—Screen Time; SD—Sleep Duration.

Figure 2 illustrates the compliance of participants with the 24-HMB guidelines at baseline. Overall, 22.0% of the young children met the PA recommendations, 35.8% met the ST guidelines, and 71.5% met the SD guidelines. Only 5.5% of the participants met all three guidelines, while 16.5% did not meet any.

### 3.2. Cross-Sectional Associations of Meeting 24-HMB Guideline Recommendations with BMD

As shown in Table 2, in the cross-sectional analysis, after adjusting for covariates including age, sex, height, weight, BMI, and SES, young children who adhered to two guidelines had a lower risk of insufficient BMD compared to those who did not meet any of the guidelines (OR = 0.34, 95% CI: 0.13, 0.88). The reduction in risk was particularly evident among young children who adhered to both the ST and SD guidelines (OR = 0.29, 95% CI: 0.09, 0.95).

### 3.3. Longitudinal Associations of Meeting 24-HMB Guideline Recommendations with BMD

Table 3 illustrates the longitudinal associations between adherence to different numbers and combinations of 24-HMB guidelines and the risk of insufficient BMD. After adjusting for age, sex, height, weight, BMI, and SES, the risk of insufficient BMD decreased with an increasing number of guidelines met at the 1-year follow-up. However, a significant reduction in risk was only observed when all three guidelines were met (OR = 0.13, 95% CI: 0.02, 0.69). Compared to young children who did not meet any guidelines, meeting the PA guideline alone at baseline (OR = 0.22, 95% CI: 0.07, 0.71), the PA and ST guidelines together (OR = 0.18, 95% CI: 0.04, 0.83), and all three guidelines (OR = 0.13, 95% CI: 0.03, 0.69) was associated with a lower risk of insufficient BMD after one year.

## 4. Discussion

This study aimed to examine the association between adherence to the 24-HMB guidelines and BMD in young children. The cross-sectional results demonstrated that children who adhered to two of the guideline recommendations had a lower risk of insufficient BMD compared to those who did not meet any recommendations. This protective effect was particularly pronounced among young children who met both the ST and SD recommendations. Longitudinally, young children who adhered to the PA guideline, both the PA and ST guidelines, or all three guidelines at baseline showed a lower risk of insufficient BMD one year later compared to those who did not meet any of the recommendations.

In this study, only 5.5% of young children met all three recommendations of the 24-HMB guidelines. This finding is consistent with research conducted in other countries, including Belgium (5.6%) [50], Canada (5.0%) [51], Singapore (5.5%) [52], Portugal (4.5%) [53], Bangladesh (4.7%) [54], Colombia (4.8%) [55], and Iran (4.8%) [56]. Research indicates that factors at the individual level (e.g., sex and age), interpersonal level (e.g., parental support and companionship), organizational level (e.g., activity arrangements in kindergartens), and community level (e.g., accessibility and safety of activity facilities) are potential influences on young children’s adherence to the 24-HMB guidelines [55,57,58]. The low adherence to the 24-HMB guidelines among young children poses a potential threat to clinical and public health outcomes. Therefore, future research should focus on developing intervention strategies aimed at improving adherence to the 24-HMB guidelines in young children. Additionally, this study found that while most young children were able to meet the SD guideline, few young children met the PA and ST recommendations. This result is consistent with previous research indicating that many young children struggle to achieve sufficient PA and limit ST [53,59]. It suggests that increasing MVPA and limiting ST in young children may have a significant impact on raising the proportion of young children who meet the 24-HMB guidelines.

With the introduction of the 24-HMB concept, research has shifted from evaluating the isolated effects of PA, ST, and SD on health outcomes to viewing them as an integrated whole [19,20]. Rollo et al. reviewed the impact of 24-HMB on health across the lifespan and highlighted the lack of research on its effects on bone health [22]. To date, studies on 24-HMB and bone health have primarily focused on children, adolescents, and older adults [60,61]. To our knowledge, this is the first study to examine the association between adherence to the 24-HMB guidelines and BMD in young children. Our findings suggest that young children who met both the ST and SD guidelines had a lower risk of insufficient BMD compared to those who did not meet any guidelines. This finding aligns with previous studies indicating that regular sleep patterns and minimized screen exposure are associated with favorable bone development outcomes [16,62]. During sedentary, screen-based behaviors, the musculoskeletal system remains unloaded, which may negatively affect bone health [63]. Additionally, bone turnover markers peak at night [64], and insufficient sleep can lead to reduced levels of bone formation markers, while bone resorption markers remain unchanged or increase, potentially leading to bone loss [65].

It is noteworthy that, despite the substantial body of research pointing to the protective effects of MVPA on young children’s bone health [10,12,66], our cross-sectional analysis did not reveal any significant associations between meeting PA-related guidelines and BMD. This may stem from the nonlinear nature of bone growth in young children, where developmental changes and genetic factors play prominent roles, potentially overshadowing short-term behavioral influences [67,68]. However, baseline PA was found to be associated with a lower risk of insufficient BMD one year later in the longitudinal analysis. This significant effect was observed whether PA was met independently, in combination with ST, or alongside both ST and SD. This suggests that MVPA may have a cumulative effect on promoting bone health in young children [12,69]. Studies have shown that PA-induced mechanical loading is an important stimulus for osteoblast differentiation and mineralization, regulating the secretion of hormones and cytokines that play a role in bone metabolism [70], and promoting bone angiogenesis through the regulation of angiogenic mediators and signaling pathways [71,72]. In simple terms, the mechanical load generated by PA stimulates osteoblast activity, thereby enhancing bone formation [73].

Our study contributes to a deeper understanding of how meeting the 24-HMB guidelines promotes health in young children, addressing the evidence gaps identified in early childhood guidelines. Furthermore, PA was objectively measured using accelerometers, and the assessment of ST included various screen-based behaviors rather than focusing solely on television watching. However, the ST and SD data for young children were reported by parents, which may have introduced recall bias and social desirability bias. Additionally, certain potential confounders that could influence young children’s BMD, such as nutritional supplements and dietary habits, were not considered in the analysis. Another limitation is the single-time assessment of PA, ST, and SD over one week to predict BMD measured a year later. This approach assumes behavior stability across the year, which may not fully capture the changes in children’s activity patterns over time. Future research could benefit from periodic assessments to better understand the cumulative effects of these behaviors on BMD. The sample was drawn solely from Changsha, Hunan, China, which may limit the generalizability of the findings to children in other cultural contexts. Unlike previous studies that assessed the isolated effects of PA, ST, and SD on young children’s BMD, our study takes an integrated approach by examining the combined effects of 24-HMB based on guideline adherence. Future research could apply compositional data analysis or cluster analysis to further explore the impact of 24-HMB on young children’s BMD, providing more comprehensive insights for public health strategies.

## 5. Conclusions

The results from our cross-sectional and one-year cohort studies suggest that adherence to the 24-HMB guidelines is associated with a lower risk of insufficient BMD in young children. Future research could initially focus on setting achievable goals, such as limiting ST and ensuring sufficient sleep, before gradually increasing MVPA and maintaining PA habits, thereby promoting BMD development in young children.

## Figures and Tables

**Table 1:** Participant characteristics (*n* = 109).

Variables	Value ^a^
Age (year)	4.3 ± 0.6
Sex	
Boy	61 (56.0)
Girl	48 (44.0)
Height (cm)	107.4 ± 7.7
Weight (kg)	18.4 ± 5.0
BMI (kg/m^2^)	
Underweight	3 (2.8)
Normal weight	88 (80.7)
Overweight	6 (5.5)
Obesity	12 (11.0)
SES	
Low	16 (14.7)
Middle	32 (29.4)
High	61 (56.0)
**24-HMB**	
LPA (min/day)	188.0 ± 40.6
MVPA (min/day)	46.7 ± 20.6
ST (min/day)	100.1 ± 91.7
SD (h/day)	11.8 ± 1.7
**BMD**	
***Baseline***	
Normal BMD	70 (64.2)
Insufficient BMD	39 (35.8)
***Follow-up***	
Normal BMD	76 (69.7)
Insufficient BMD	33 (30.3)

^a^ Values are mean ± SD or n (%). Abbreviations: BMI—Body Mass Index; SES—Socioeconomic Status; 24-HMB—24-Hour Movement Behavior; LPA—Light Physical Activity; MVPA—Moderate-to-Vigorous Physical Activity; ST—Screen Time; SD—Sleep Duration; BMD—Bone Mineral Density.

**Table 2:** Cross-sectional associations of meeting 24-HMB guideline recommendations with BMD.

Variables (T1)	BMD (T1)	
	OR (95% CI)	*p*
The number of recommendations met		
Zero	Ref	Ref
One	1.38 (0.61, 3.12)	0.445
Two	0.34 (0.13, 0.88)	0.026
Three	3.49 (0.56, 21.61)	0.180
24-HMB combinations		
Meeting none	Ref	Ref
Only PA	1.43 (0.11, 18.18)	0.781
Only ST	1.35 (0.32, 5.74)	0.686
Only SD	1.25 (0.52, 3.00)	0.625
PA + ST	2.36 (0.54, 10.36)	0.255
ST + SD	0.29 (0.09, 0.95)	0.041
PA + SD	0.73 (0.20, 2.66)	0.632
PA + ST + SD	3.49 (0.56, 21.61)	0.180

Notes: All models were adjusted for age, sex, height, weight, BMI, and SES. Abbreviations: BMI—Body Mass Index; SES—Socioeconomic Status; 24-HMB—24-Hour Movement Behavior; ST—Screen Time; SD—Sleep Duration; BMD—Bone Mineral Density.

**Table 3:** Longitudinal associations of meeting 24-HMB guideline recommendations with BMD.

Variables (T1)	BMD (T2)	
	OR (95% CI)	*p*
The number of recommendations met		
Zero	Ref	Ref
One	0.97 (0.38, 2.47)	0.948
Two	0.94 (0.36, 2.47)	0.905
Three	0.13 (0.02, 0.69)	0.016
24-HMB combinations		
Meeting none	Ref	Ref
Only PA	0.22 (0.07, 0.71)	0.011
Only ST	2.32 (0.91, 5.91)	0.079
Only SD	1.00 (0.36, 2.73)	0.993
PA + ST	0.18 (0.04, 0.83)	0.028
ST + SD	1.92 (0.72, 5.11)	0.192
PA + SD	3.07 (0.97, 9.67)	0.055
PA + ST + SD	0.13 (0.03, 0.69)	0.016

Notes: All models were adjusted for age, sex, height, weight, BMI, and SES. Abbreviations: BMI—Body Mass Index; SES—Socioeconomic Status; 24-HMB—24-Hour Movement Behavior; ST—Screen Time; SD—Sleep Duration; BMD—Bone Mineral Density.

## Data Availability

Data may be made available from the first author (lidan97@hunnu.edu.cn) on reasonable request.

## Abbreviations

24-HMB = 24-Hour Movement Behavior; LPA = Light Physical Activity; MVPA = Moderate-to-Vigorous Physical Activity; ST = Screen Time; SD = Sleep Duration; BMD = Bone Mineral Density; BMI = Body Mass Index; SES = Socioeconomic Status; OR = Odds Ratio; 95% CI = 95% Confidence Interval.
